# Congenital amusia—pathology of musical disorder

**DOI:** 10.1007/s13353-021-00662-z

**Published:** 2021-09-21

**Authors:** Krzysztof Szyfter, Jadwiga Wigowska-Sowińska

**Affiliations:** 1 Institute of Human Genetics of the Polish Academy of Sciences, Ul. Strzeszyńska 32, 60-479 Poznań, Poland; 2 Department of Developmental Neurology, Poznań University of Medical Sciences, Poznań, Poland

**Keywords:** Congenital amusia, Pathophysiology, Disconnection syndrome, Hereditary disorder

## Abstract

Amusia also known as tone deafness affects roughly 1.5% population. Congenital amusia appears from birth and lasts over life span. Usually, it is not associated with other diseases. Its link to hearing impairment has been definitively excluded. Neurobiological studies point to asymmetrical processing of musical signals in auditory cortex of left and right brain hemispheres. The finding was supported by discovering microlesions in the right-side gray matter. Because of its connection with asymmetry, amusia has been classified to disconnection syndromes. Alternatively to the neurobiological explanation of amusia background, an attention was turned to the significance of genetic factors. The studies done on relatives and twins indicated familial aggregation of amusia. Molecular genetic investigations linked amusia with deletion of 22q11.2 chromosome region. Until now no specific genes responsible for development of amusia were found.

There are two extreme types of music perception. The first is absolute pitch which means that a sound is being perfectly identified without any reference such as tuning fork or piano key. The studies on absolute pitch have established its background derived both from genetic and environmental factors. Interesting ethnic differences, favoring Far East Asia inhabitants who use tune languages, were reviewed (Szyfter and Witt 2020).

The opposite situation is connected with the total insensitivity to music which is recognized simply as an unorganized noise. A fully developed congenital amusia is characterized by a deficit of perception of tune, melody, and rhythm, which together form a structure of music. A formal term in use to define amusia (congenital or acquired) is “tonal deafness,” used in connection to both perception and production of music.

An estimation of amusia prevalence in Western world, based on self-reported questioners or a simple test, has suggested 4% amusic individuals in a random population. Further studies have established special tools (the most common are: Montreal Battery for Evaluation of Amusia /MBAE/and Distorted Tune Test /DTT/) to make amusia a measurable condition (Peretz 2008). An application of such quantitative techniques used in testing 20,850 Canadian participants has provided an estimation of the prevalence of congenital amusia of 1.5%. The latter result is lower than the old results from eighties of the XX century. Amusia was found to be slightly more common in females that in males (Peretz & Vuvan 2017). Another recent study, done on 383 Colombian university students, established a prevalence of pitch-based amusia to be as low as 0.52% (Pradilla et al. 2020).

## Phenotypic description of amusia

A number of studies attempted to reveal precisely a phenotype of amusic individuals. A disability to recognize musical tunes is a persisting feature. This deficit extends to music memory (Ayotte et al. 2002). The studies done on Caucasians have shown that difficulties do not affect language domain (Williamson and Stewart 2013). The latter observation, however, is not applicable for users of tone languages such as Mandarin Chinese, Japanese, and Vietnamese. It has been shown that individuals speaking these languages and diagnosed for amusia were worse than controls in both speech and music imitation. The defect was stronger in music than speech processing (Liu et al. 2013).

It was observed that deficit in the perception of pitch and rhythm co-occurred in amusic individuals. Rhythm perception impairment was explained by a deficit of basic timekeeping ability (Tranchant & Peretz 2020). The studies performed on a relatively large group of mono and dizygotic Finnish twins suggested a genetics nature of pitch recognition and more likely environmental factor in the deficit of rhythm perception (Seesjärvi et al. 2016). However, a small part of affected individual preserved a good beat perception which allowed for a limited participation in such music performance as choral singing or playing instruments in an amateur music band (Lagrois and Peretz 2019).

Congenital amusia is usually recognized in children having normal intellectual and language skills. It was observed that amusia is connected predominantly with the deficit in detecting small pitch changes but incorrect rhythm detection is less severly affected (Lebrun et al. 2012). It is assumed that amusia manifests from birth. In connection with this assumption, it was shown that daily music listening does not help and amusia persists (Goulet et al. 2012). Noteworthy, it was found that musical emotions could be recorded in amusical individuals independently on their impaired recognition of pitch and rhythm (Leveque et al. 2018).

Then, a question emerged, whethers amusia is a single disorder or is it associated with other symptoms. It has been firmly established that amusia is not derived from hearing loss, brain damage, or intellectual deficiencies. Only weak associations with dyslexia (Couvignou and Kolinsky 2021), syntactic processing in music and language (Sun et al. 2018), and spatial orientation [14] were found. The study of Couvignou and Kolinsky (2021) testing 38 dyslecting children by MBAE test has revealed amusia in 34% of the study group versus 5% amusicals in the typically developing children. All the mentioned symptoms are only marginally identifiable in amusical subjects.

However, it was supposed that the disability of music learning could be considered as a defect of cognition ability in a broad sense. That in turn raised the question of intelligence of amusical persons (Mosing et al. 2014). Such connection has been definitively excluded based on two lines of evidence. Language acquiring, which is another important cognitive function, in amusicals was slow only at early phase to get normalized later on (Ayotte et al. 2002; Sun et al. 2018). Second, an intellectual development in amusicals proceeded fully normally without any limitations. Finally, looking at a few notable persons affected by amusia such as pope Francisco, Che Guevarra, Theodore Roosevelt, Sigismund Freud, and Charles Darwin (Ayotte et al. 2002, Williamson & Stewart 2013, Peretz 2008), one could consider their life success as a definitive proof of intelligence. Another example worth to mention is the case of Maurice Ravel (1875–1937), a famous French composer, conductor, and pianist. He has suffered from amusia in the last 5 years of his life, as a consequence of car accident. That time, the impairment was not classified as a disease but now it would be diagnosed as acquired amusia (remaining outside the frame of the review) (Vitturi & Sanvito 2019).

Altogether, congenital amusia is present from birth and persists lifelong. It seems to be a single music-specific defect in an affected individual. To understand the biological bases of amusia, studies have been undertaken in two directions: neurobiology and genetics (Gingras et al. 2015).

## Neurobiological background

It was established that musical input passes cochlea, brainstem, primary auditory cortex to reach superior temporal gyrus. Sound waves are being transformed in inner ear into electric signals. Along properly working pathways, the signal is undamaged but in a musical brain the problem begins in the auditory cortex because of impaired pitch perception in between the left and right hemispheres. A reduced connectivity between the left and right auditory cortex was established in the brain of amusic individuals. It means that properly recruited music signals are processed separately in both hemispheres and the dual stream of signals is not analyzed in the brain jointly (Peretz 2016).

**Fig. 1:**
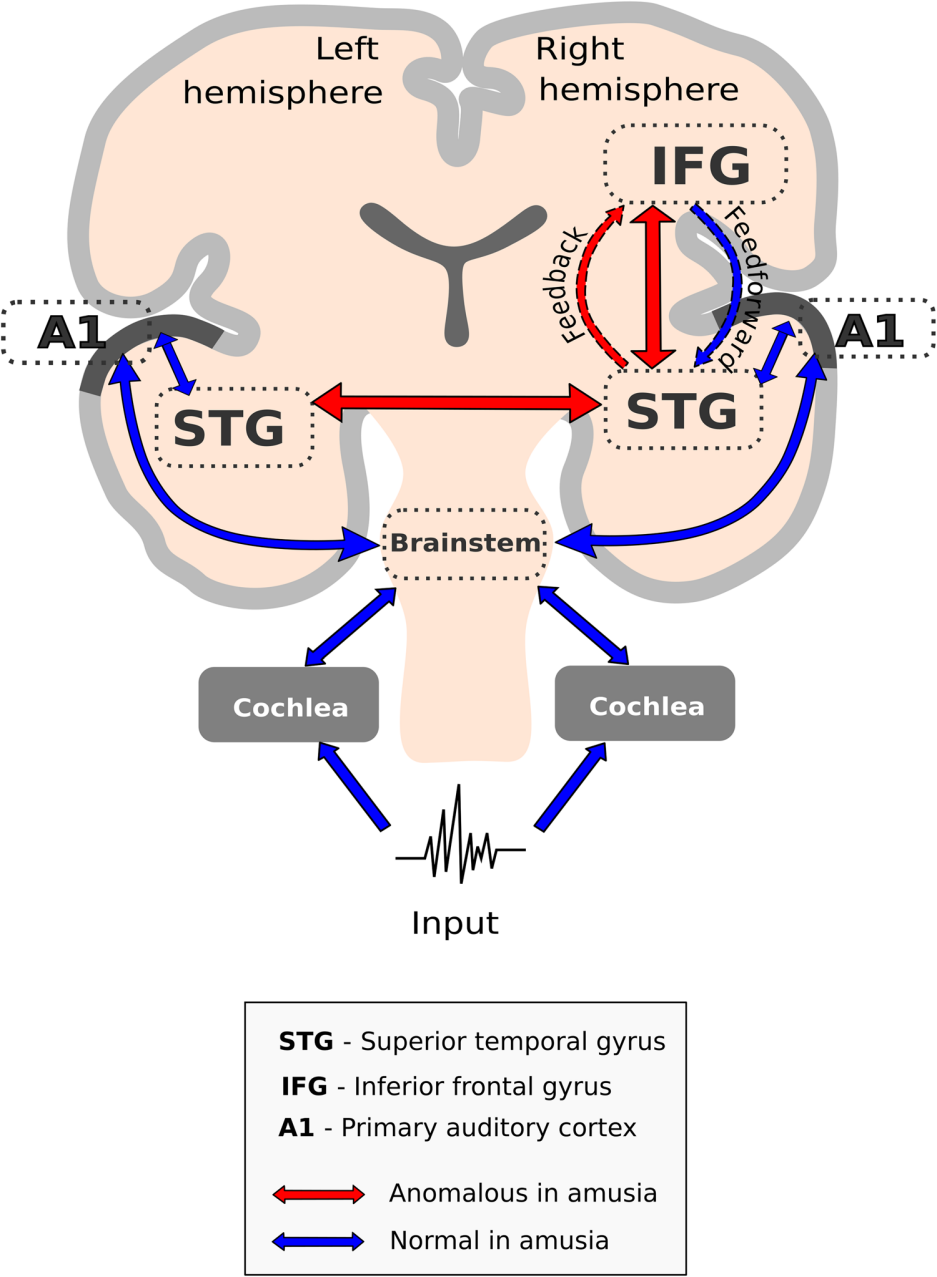
Final stage of signal analysis in the auditory cortex

To reach a better understanding of neurological aspect of amusia, microstructural changes were investigated in 12 subjects with congenital amusia and compared with 20 matched controls. Amusics have been shown to have higher diffusivity indices in the corpus callosum (white matter linking the cerebral cortex of the left and right cerebral hemispheres) and the right occipital fasciculus (white material running vertically in the rear of the brain). The axial diffusivity in the latter structure was negatively correlated with musical scores in the amusia group. All these findings imply for recognizing congenital amusia as a disconnection syndrome (Wang et al. 2017). Disconnection was studied by magnetoencephalography in two groups of participants listening music. The study has shown a decreased intrinsic connectivity within auditory cortex in both hemispheres, increased lateral connectivity between auditory cortices, as well as a decreased right fronto-temporal backward connectivity in amusics as compared with controls. Voxel-based morphometry revealed anomalies in white and gray matter concentrations in the right inferior frontal gyrus and the right superior temporal gyrus in the amusic brain (Albouy et al 2013). Another study tested a response to harmonic musical syntactic structures in 16 amusical persons compared with 16 matched controls. The study has demonstrated a similar reaction for the early detection stage. The differences appeared in the later integration stage confirming hierarchical processing of the music. Hence, violation of music perception is taking place at the final stage of signal analysis in the auditory cortex (Fig. 1). A break of the interhemispheric fibers was considered as the cause of hemispheric disconnection [Peretz 2016, Zhou et al 2019].

## Significance of genetic factor

The studies on the role of genetic factor as contributor to amusia were mostly performed in frame of classical Mendelian genetics. The early observations on congenital amusia prevalence in families were later on confirmed by regular investigations documenting familial aggregation of this behavioral defect. A large study (over 20,000 cases) by Peretz and Vuvan (2017) cited above shed more light on amusia prevalence in families. Forty-six percent first-degree relatives of affected persons were carriers of this defect. Another study of I. Peretz laboratory (Montreal, Canada) using MBAE test in 71 members of 9 large families established that 39% of first-degree relatives have the same cognitive disorder. Musical training did not help affected persons (Peretz et al. 2007). Another study using the same method in 105 amusia youths (mean age 12.5 years) has also shown music difficulties to be stronger in children whose father suffered for amusia (Wilcox et al. 2015).

The further progress in understanding congenital amusia pathophysiology was achieved in twin studies. An early study was performed on 148 dizygotic and 136 monozygotic Caucasian twin pairs using DTT for testing musical pitch recognition (Drayna et al. 2001). The authors did not select amusia individuals, dealing instead with subjects representing the full scope of music perception from absolute pitch carriers to amusicals. The conclusion derived from the study was that musical pitch recognition is primarily due to a highly heritable auditory function with almost ignorable impact of shared environment. The latter conclusion was strongly confirmed by the study exploring music perception in 10,500 Swedish twin pairs (Mosing et al. 2014). Music discrimination was studied separately with respect to connection to pitch, melody, and rhythm. Similarity of the studied parameters in both studies was explained by shared genetic influences. The study attempted further to correlate music discrimination with intelligence estimated by IQ coefficient determined by a visual matrix test. The results of the study suggested moderate positive associations between different musical auditory discrimination tasks. The authors concluded that genetic impact on musical discrimination involves in part genes that influence not only musical but also non-musical cognitive tasks. Covariation of these two factors was explained by the genetic pleiotropy (Mosing et al. 2014). The aim of another study by Finnish researchers was to investigate an impact of genetic on the (separately) pitch, melody, and rhythm factor. The study by MBEA test comprised 384 young and adult mono- and dizygotic twins. The study demonstrated again a high genetic impact on pitch and melody recognition deficits. Rhythm recognition appeared to have stronger dependence on the environmental factor (Seesjärvi et al. 2016). It would be of interest to cite a finding of a 27-year-old female twin pair with one amusic twin with the sister having normal pitch and rhythm perception (Pfeifer and Haman 2018).

Molecular genetic studies of the significance of genetic factor in congenital amusia did not largely reach chromosome or gene level. The only paper in the field indicated for a possible association of amusia with the deletion of 22q11.2 chromosome region (Gao et al. 2014). An association of 22q11.2 deletion syndrome has already been linked to the impairment of cognitive ability and several psychiatric disorders. Fifty-eight individuals aged 8–29 years with 22q11.2 deletion syndrome were recruited from another study and tested by DTT. Individuals with this chromosome aberration had a significant impairment of musical auditory processing. At the same time, the presence of any psychiatric diagnoses was excluded in the study group. Altogether, pitch discrimination as a part of cognitive ability seems to be linked to the deleted chromosome band, but remains independent on psychiatric illness.

The lack of reports on particular genes involved in congenital amusia seems to be confusing, especially since genes determining opposite situation as perfect pitch were already recognized. Such genes as *AVPR1A* on chromosome 12 implicated in music perception and attributed to perfect pitch carriers, gene *SLC6A4* on chromosome 17q was associated with music memory or several loci on chromosome 4 connected with singing, and music perception were identified (Tan et al. 2014). Whether the same genes are impaired (deleted? mutated? dysregulated by other mechanisms?) remains an open question. In any case, aggregation and twin studies suggestively indicate for a significance of genetic factor in amusia.

Taking into account all presented findings, the phrase “the music of the genes” (Jobling 2014) reflects the need for further investigations.
